# Re-evaluation of HER2 status in 606 breast cancers—gene protein assay on tissue microarrays versus routine pathological assessment

**DOI:** 10.1007/s00428-020-02768-x

**Published:** 2020-02-20

**Authors:** Emma Sandén, Somayeh Khazaei, Helga Tryggvadottir, Signe Borgquist, Karolin Isaksson, Karin Jirström, Helena Jernström

**Affiliations:** 1 Department of Clinical Sciences in Lund, Oncology and Pathology, Faculty of Medicine, Lund University Cancer Center / Kamprad, Lund University and Skåne University Hospital, Lund, Sweden; 2 Departments of Oncology and Clinical Medicine, Aarhus University and Aarhus University Hospital, Aarhus, Denmark; 3 Department of Clinical Sciences in Lund, Surgery, Faculty of Medicine, Lund University Cancer Center, Lund University and Skåne University Hospital, Lund, Sweden, and Central Hospital Kristianstad, Kristianstad, Sweden

**Keywords:** HER2, Tissue microarray, Whole tissue section, Immunohistochemistry, In situ hybridization, Gene protein assay

## Abstract

Human epidermal growth factor receptor 2 (HER2) status in breast cancer is routinely determined through immunohistochemistry (IHC) and/or in situ hybridisation (ISH) performed on whole tissue sections (WS). The purpose was to evaluate whether a gene protein assay (GPA) combining IHC with ISH, performed on breast cancer tissue microarray (TMA), is suitable for large-scale retrospective HER2 status evaluation. TMAs from 606 tumours from a Swedish population-based cohort (2005–2012) were stained with GPA. GPA IHC on TMA yielded weaker staining than IHC on WS during routine pathological assessment (86.0% agreement). However, final HER2 status agreement between GPA on TMA and WS based on both IHC and ISH was 97.7%. Only 14 tumours were discordant and one tumour with IHC score 1+ on both TMA and WS was *HER2* amplified on TMA. In conclusion, GPA on TMA is suitable for large-scale retrospective evaluation of HER2 status.

## Introduction

Human epidermal growth factor receptor 2 (HER2) is overexpressed in 15–20% of breast cancers, predominantly due to gene amplification. In clinical breast cancer diagnostics, this biomarker guides treatment selection, as HER2 overexpression predicts response to targeted therapies [1].

In routine clinical diagnostics, HER2 status is determined through immunohistochemistry (IHC) to detect protein overexpression and in situ hybridization (ISH) to confirm gene amplification. According to Swedish guidelines [2], whole tissue sections (WS) should initially be evaluated with IHC. Membrane staining intensity and fraction of positive cells determine a HER2 IHC score between 0 and 3+. Tumours with IHC scores 0 or 1+ are considered HER2-negative and WS are not further analysed for gene amplification. IHC scores of 2+ or 3+ suggest *HER2* amplification and complementary ISH is then recommended. Gene amplification may therefore be missed in tumours with low protein levels.

Tissue microarrays (TMAs) are constructed from tumour cores and are commonly utilized for retrospective analyses of large cohorts. In 2005, the first HER2 gene protein assay (GPA) was introduced [3], and the feasibility of staining TMAs with GPA was first demonstrated in 2014 [4]. GPA allows for simultaneous gene and protein staining on the same section. It is cost-efficient, time- and tissue-saving and potentially diagnostically advantageous [5]. To our knowledge, there are no studies comparing GPA on TMA for retrospective HER2 analysis with HER2 status obtained during routine pathological assessment on WS.

The primary aim of this study was to investigate whether GPA on TMA is suitable for large-scale retrospective evaluation of HER2 status. The second aim was to elucidate whether *HER2* gene amplification was present in tumours with IHC scores 0/1+ and not detected during routine pathological assessment.

## Methods

This study is based on a subcohort of patients from the population-based BC-blood study [6]. Between November 2005 (when HER2 status was incorporated into routine pathological assessment) and June 2012, 738 patients with primary invasive breast cancer and no preoperative treatment were included at Skåne University Hospital in Lund, Sweden. HER2 status was available for 689 patients. Of these, 600 patients with a median age of 61 years (range 24–88 years) had 606 tumours (6 bilateral) that were evaluable on TMA. Of these 606 tumours, 427 were ≤ 20 mm and 179 were > 20 mm or had muscular/skin involvement; 181 were histological grade III; 504 were mainly of no special type (formerly ductal), 65 mainly lobular and 37 of other/mixed histology; 366 were node-negative, 527 oestrogen receptor-positive and 426 progesterone receptor-positive.

HER2 status obtained during pathological diagnostics was retrieved from pathology reports. According to clinical routine in Lund, Sweden, IHC with HercepTest (DAKO K5206, Copenhagen, Denmark) in 2005–2010 and as of 2011 HER-2/neu, PATHWAY Ventana 790-2991 (Ventana Medical Systems) and/or fluorescent ISH (FISH) with *HER2* FISH pharmDx™ Kit (DAKO K5331) was performed on WS. Between 2005 and 2009, HER2 positivity was defined as FISH *HER2*/CEP17 ratio > 2, or FISH *HER2* copies > 6. The IHC cut-off was 10% and IHC 2+ and 3+ tumours were analysed with FISH [7]. Between 2010 and 2012, HER2 positivity was defined as either > 30% IHC 3+, or ISH *HER2*/CEP17 ratio > 2.2, or ISH *HER2* copies > 6, in analogy with St Gallen guidelines [8].

Dual 1 mm cores from representative invasive tumour regions (selection based on H&E staining) of formalin-fixed paraffin-embedded tissue blocks were collected from surgical specimens. The cores were assembled into TMAs, using a semi-automated tissue array device (Beeches instruments, Sun Prairie, WI). TMA HER2 status was evaluated with IHC and dual ISH (DISH) using a double gene and protein staining protocol [9]. The antibody PATHWAY anti-HER-2/neu (4B5; Ventana Medical Systems) and INFORM HER2 Dual ISH DNA Probe Cocktail 800-422 (Roche Tissue Diagnostics) were used. TMA HER2 positivity was defined as either IHC 3+ or DISH *HER2*/CEP17 ratio ≥ 2. According to current Swedish guidelines, HER2 positivity is defined as either IHC3+, or 2+ and ISH *HER2*/CEP17 ratio ≥ 2, or IHC2+ and ISH *HER2* copies > 4 [2]. The staining was evaluated by two independent readers (E.S. and S.K.) blinded to clinical data. In case of discrepancy between the readers, a re-evaluation was made and consensus was reached. A pathologist (K.J.) was consulted for 15 tumours.

Concordance between readers, and between WS and TMA, was assessed with agreement (%) and Cohen’s κ. Disagreement was analysed with McNemar’s test (GraphPad Prism version 8.00, GraphPad Software, La Jolla California USA, www.graphpad.com).

## Results

**Fig. 1:**
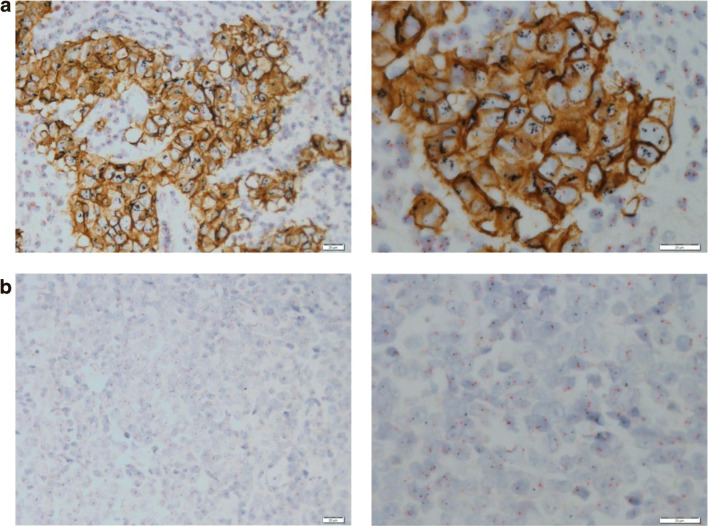
Representative images of GPA staining of TMA cores. **a** IHC 3+, amplified. **b** IHC 0, non-amplified. Scale bars are 20 μm

Figure 1a–b shows representative images of double gene and protein staining of HER2-positive and negative TMA cores. The concordance between the two readers was good for IHC (89.9%; κ = 0.73; 95% CI 0.66–0.79) and very good for the final HER2 status (97.7%; κ = 0.89; 95% CI 0.83–0.95).

Since Swedish guidelines recommend complementary ISH analyses in tumours with IHC 2+ and 3+, the concordance between TMA and WS regarding negative (0 or 1+) and intermediate or high (2+ or 3+) IHC scores was assessed (Table 1). Five cases had missing IHC status on WS, leaving 601 tumours. The agreement between the IHC scores on TMA and WS (0/1+ vs. 2+/3+) was good (86.0%; κ = 0.61; 95% CI 0.53–0.68). For the 84 discordant pairs, the odds for a negative IHC staining were significantly higher on TMA compared with WS (OR 1.71; 95% CI 1.08–2.76; McNemar’s test *P* = 0.022).

**Table 1:** HER2 IHC score and final HER2 status (IHC and ISH) in WS and TMA

		TMA HER2 IHC	
		0/1+	2+/3+
WS HER2 IHC	0/1+	420	31
	2+/3+	53	97
		TMA HER2 status	
		Neg	Pos
WS HER2 status	Neg	530	9
	Pos	5	62

The results for FISH on WS and DISH on TMA were then incorporated to render the final HER2 status. Chromosome 17 staining was missing in 12 TMA tumours; three had IHC 3+ and were classified as positive, whereas nine had IHC scores 0/1+ and were classified as negative. The agreement between TMA and WS was very good (97.7% κ = 0.89; 95% CI 0.83–0.95). None of the tumours with missing chromosome 17 staining on TMA had discordant HER2 status between WS and TMA.

For the 14 discordant pairs, the odds for a positive HER2 status was non-significantly higher when obtained from TMA compared with WS (OR 1.80; 95% CI 0.54–6.84; McNemar’s test *P* = 0.42). In five of the 14 tumours, HER2 status was only positive when obtained from WS. For the remaining nine samples, HER2 status was only positive on TMA. There were between one and four tumours with discrepant HER2 status per year (2005–2012).

For one tumour, the IHC score was 1+ with both methods and the DISH of the TMA showed gene amplification. No FISH had been carried out during routine pathological assessment.

## Discussion

This is the first study that compares HER2 status obtained by GPA on TMA with routine pathological assessment. The results showed a very good agreement between the methods. This is consistent with previous studies comparing agreements between HER2 status on WS and TMA using IHC and ISH as single assays [10, 11]. In the current study, the agreement between IHC scores was somewhat lower than for the final HER2 status. In multicentre comparisons of HER2 analyses, the IHC scores are more often discordant than the ISH results [12, 13]. Potential explanations for discrepancies between IHC scores in retrospective analyses, including the current one, could be interobserver variability, different antibodies, changes in IHC cut-offs and the influence of long-term storage on the HER2 epitope. Moreover, HER2 protein levels captured by IHC may be more heterogeneous than gene amplification across the tumour [14].

In the current study, the odds for weaker IHC staining on TMA compared with WS were nearly two-fold for discordant tumours. Further, chromosome 17 staining was missing for a few tumours. Although not seen in this study, the weaker IHC obtained by GPA could potentially lead to misclassification of tumours where chromosome 17 staining is missing.

Nine discordant tumours in this cohort displayed simultaneous discrepancy of IHC scores and FISH/DISH results between the WS and TMA (data not shown), suggesting a heterogeneous HER2 expression, which has been previously demonstrated by others [15]. As TMA cores only represent a small part of the tumour, some discrepancy is expected. There were some changes in the Swedish HER2 assessment guidelines 2005–2012, while all TMAs were evaluated according to current guidelines [2]. The discordant samples were distributed across all years. Guideline changes are thus unlikely to explain the discrepancies.

According to current Swedish guidelines [2], ISH is only recommended for tumours with an IHC 2+ or 3+. The frequency of false-negative breast tumours (i.e. IHC 0/1+ with *HER2* amplification) in breast cancer patients in Sweden is therefore poorly assessed. Retrospective international studies have indicated that such an approach may overlook a small number of amplified breast tumours, harbouring aggressive features such as higher grade and Ki67 expression [16]. In the current analysis, only one tumour with IHC 1+ on both TMA and WS was *HER2* amplified on TMA. Hence, our results do not support routine ISH for cases with negative IHC staining.

In conclusion, GPA on TMA is suitable for retrospective analysis of HER2 status. Dual protein and gene staining tentatively yield a weaker IHC staining than IHC alone. However, the overall agreement between routine pathological assessment and GPA on TMA was very good when DISH was taken into consideration. Moreover, the results do not support performing ISH for all samples irrespective of IHC score obtained during routine pathological assessment.
